# Trends in Passive IoT Biomarker Monitoring and Machine Learning for Cardiovascular Disease Management in the U.S. Elderly Population

**DOI:** 10.20900/agmr20230002

**Published:** 2023-03-31

**Authors:** Brian F. Bender, Jasmine A. Berry

**Affiliations:** 1 Intake Health Inc., Raleigh, NC 27601, USA; 2 Robotics Institute, University of Michigan, College of Engineering, Ann Arbor, MI 48109, USA

**Keywords:** internet of things (IoT), geriatric, cardiovascular diseases, machine learning, passive monitoring, artificial intelligence, hypertension, heart failure, CVD, remote care

## Abstract

It is predicted that the growth in the U.S. elderly population alongside continued growth in chronic disease prevalence will further strain an already overburdened healthcare system and could compromise the delivery of equitable care. Current trends in technology are demonstrating successful application of artificial intelligence (AI) and machine learning (ML) to biomarkers of cardiovascular disease (CVD) using longitudinal data collected passively from internet-of-things (IoT) platforms deployed among the elderly population. These systems are growing in sophistication and deployed across evermore use-cases, presenting new opportunities and challenges for innovators and caregivers alike. IoT sensor development that incorporates greater levels of passivity will increase the likelihood of continued growth in device adoption among the geriatric population for longitudinal health data collection which will benefit a variety of CVD applications. This growth in IoT sensor development and longitudinal data acquisition is paralleled by the growth in ML approaches that continue to provide promising avenues for better geriatric care through higher personalization, more real-time feedback, and prognostic insights that may help prevent downstream complications and relieve strain on the healthcare system overall. However, findings that identify differences in longitudinal biomarker interpretations between elderly populations and relatively younger populations highlights the necessity that ML approaches that use data from newly developed passive IoT systems should collect more data on this target population and more clinical trials will help elucidate the extent of benefits and risks from these data driven approaches to remote care.

## INTRODUCTION

Age is arguably among the most significant health determinants and is a risk factor for various human pathologies, especially those concerning the heart. Cardiovascular Diseases (CVD), which includes coronary heart disease, stroke, heart failure and peripheral artery disease, are leading causes of morbidity and mortality in U.S. adults and is expected to continue to rise in the future [1]. During this same projected timeframe, the U.S. Census Bureau estimates that the elderly population, those aged 65 and older, will rise from roughly 15% of the population today to nearly 25% by 2060 [2]. This age demographic once accounted for 23% of the total disease burden across the globe with approximately half of that burden belonging to high-income countries such as the U.S. [3]. This rise will strain an already burdened healthcare system that is struggling to provide equitable care across the population and meet this growing demand [4].

Technological advantages within the category of “Internet-of-Things” (IoT) has enabled passive and remote monitoring of data collected across various industries and applications due to ubiquitous internet connectivity, advances in the miniaturization of wireless hardware technologies, and improvements in a wide variety of sensor technologies. Naturally, these advancements have started to emerge in healthcare-specific applications [5]. These technologies offer the potential for a variety of applications such as lifestyle management and disease prevention, disease screening, disease diagnosis, and treatment management. IoT technologies have promise in personalized and preventive care because they are capable of collecting health data at a much higher frequency than during bespoke doctor visits. The increasing trend of passivity among IoT devices, whereby data is collected “in the background” and does not require user input, greatly increases both adoption and quantity of health data [6]. Subsequently, as more data becomes available, machine learning (ML) approaches become useful for applying that data towards new and improved healthcare management tools [7].

The adoption of home health technologies among the elderly population has been increasing in recent years [8]. This is due to several factors such as the aging population, advancements in technology, and the need to reduce healthcare costs. According to a survey conducted in 2023 by Rock Health [8], the use of telemedicine among older adults has increased by 12% in the past year to 76% while 21% of older adults reported they use wearable technology to manage their health, following a steady increase year over year from 13% in 2019. Nevertheless, there are still barriers to adoption for some older adults, such as lack of access to technology, lack of digital literacy, and lack of trust in technology.

**Figure 1.**
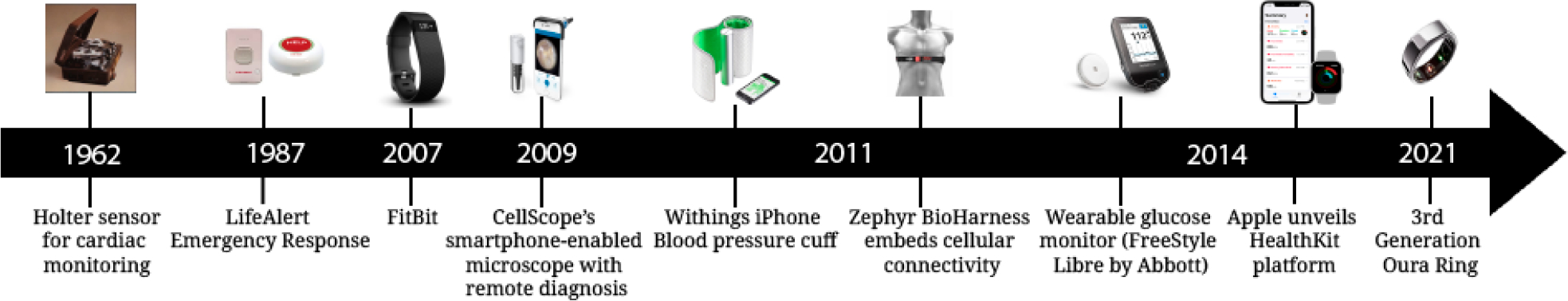
Timeline history of the progression of medical device products. *Image credit (left to right): National Museum of American History; Life Alert Emergency Response Inc.; Alphabet Inc.; CellScope Inc.; Nokia Inc.; Medtronic Inc.; Abbott Inc.; Apple Inc., Oura Inc.

The digitization and consumerization of healthcare is taking disease management along a new paradigm, whereby advanced data analysis techniques that use ML enable more personalized and real-time lifestyle and disease management care in addition to new applications altogether like predictive insights of pending events. These advances come as a consequence of an increase in device connectivity, portability, and tracking passivity which leads to an increase in more data followed an increase in ML capabilities and model training across wider healthcare applications (Figure 1). For example, the Apple Watch’s continuous accelerometer data was recently used and FDA-cleared for monitoring tremors and dyskinesia in Parkinson’s patients [9], and the Whoop wristband recently demonstrated the ability to predict preterm birth using heart rate variability (HRV) data [10]. The application of these ML algorithms require large, personalized datasets. These datasets are best acquired through continuous patient monitoring using passive collection techniques that minimize or eliminate user collection burden. This mini-review aims to highlight the recent developments in passive, IoT-based biomarker data collection that targets the elderly population’s needs within the context of CVD. We will address the opportunities and challenges that emerge at the intersection of IoT technologies and healthcare needs.

## PASSIVE IoT MONITORING FOR ELDER CARE

### Dynamic, Non-Invasive CVD Biomarkers

Primary risk factors for CVD include diabetes, dyslipidemia, hypertension, and obesity [1]. The prevalence of hypertension alone is estimated to be roughly 75% for adults over the age of 60 [11]. Measuring and monitoring hypertension is typically done through the use of traditional biomarkers such as blood pressure, heart rate, and cholesterol levels. Some examples of emerging biomarkers for measuring and monitoring hypertension, CVD events, and CVD progression over time that are the target of IoT technologies include analytes of interest within physiological fluids like blood, plasma, urine, sweat, saliva, and tears such as N-terminal pro-B-type natriuretic peptide (NT-proBNP) [12], high-sensitivity troponin (hsTnT) [13], C-reactive protein (CRP), interleukin-6 (IL-6) [14], urine albumin and creatinine [15], urine sodium and potassium [16], urine cystatin-C [17], and an array of microRNAs have all been found to be associated with hypertension and cardiovascular disease [14]; aortic stiffness which can be measured through pulse wave velocity [18]; and an ever-growing array of digital biomarkers derived from new algorithms that interpret existing data in new ways, such as the use of HRV, BCG variability, and glucose variability and their correlations to hypertension and heart failure, among others [19–22].

### Passive Sensor Technologies

Sensor *passivity* refers to the degree to which sensors can collect data without user intervention and can range from items with small active interventions like stepping on a scale (whereby longitudinal data is processed in the background) to devices that have eliminated user intervention such as mains-powered connected smart furniture. This mini review examines the passivity of IoT technologies because the high-frequency, long-term data collection advantages of passive devices enable more sophisticated ML approaches to biomarker discovery and support throughout the treatment journey, especially among the geriatric population where active use of technology can be more challenging [23].

Wearable technologies are now available with a wide array of miniaturized sensors that are capable of collecting high-frequency data and used for CVD risk classification, prevention, diagnosis, and treatment management [24] and have advantages over traditional heart monitoring methods due to their ability to be integrated into passive frameworks and provide continuous monitoring [25]. Photoplethysmography (PPG) is a non-invasive optical technique used in most wrist-based wearable devices that measures changes in light absorption or reflection as a result of blood volume changes in the microvascular bed of tissues [26]. Electrocardiography (ECG) is a medical test that measures the electrical activity of the heart and is obtained by placing electrodes on the skin. Ballistocardiography (BCG) is a non-invasive measurement technique that has been used to measure the mechanical forces generated by the beating of the heart, breathing activity, and sleep [27], and accelerometers and gyroscopes collect motion data that has been linked to CVD risk [28].

Continuous glucose monitors are growing in use among individuals with diabetes as a means for passive blood sampling and have improved care for many Type 1 and Type 2 diabetics [29]. However, in addition to applications in diabetes, glucose variability is correlated to CVD-related biomarkers of arterial stiffness and micro- and macrovascular complications [30–32] along with blood pressure variability [21,22]. A variety of analyte-specific biochemical analysis techniques, often employing electrochemical sensing, are also being miniaturized and becoming internet-connected and housed within passive testing systems [33].

### Passive IoT Integration

Smartwatches and other wrist-borne devices offer some of the most widely tested and adopted technologies for health and wellness monitoring [34]. In addition to acute care applications like fall detection, CVD biomarker monitoring applications are emerging. For example, accelerometer-based activity monitoring has shown to be useful for predicting total CVD incidence, stroke, and coronary heart disease among adults with hypertension [28] as well as heart failure and Type 2 diabetes incidence [35]. Patch-like wearables worn on the skin collecting ECG data offer high-quality cardiac monitoring for a variety of arrhythmias [36], but heart rate monitoring capabilities of smartwatches are improving quickly and in use for detection of atrial fibrillation and preferred over patch-based systems among older adults [37]. HRV has grown in use for wellness and fitness applications but is also emerging as a potential tool for chronic disease monitoring. HRV provides a potential biomarker for hypertension and CVD risk and treatment monitoring [38]. Yet, as some studies still demonstrate, digital biomarkers derived from heart rate and HRV data must be taken from the elderly populations as age-related differences are likely [39].

Clothing represents an opportunity for passive monitoring and can provide localized sensor placement beyond the wrist. For example, smart socks have been tested for peripheral neuropathy [40], as well as shoe insoles that passively and continuously measure temperature and pressure [41]. More recently, the application of ML classification algorithms for the purpose of predicting risk of diabetic foot ulcers has been used on this data for real-time risk assessment with high levels of accuracy [42]. heart rate monitoring technologies have been similarly explored among socks as well as shirts and other garments [43]. These implementations still face barriers to adoption, however, because they are often still overly bulky, require frequent battery recharging, and have relatively high costs [23,37,43].

Integrating sensors into furniture and other structures used within the home is another increasingly explored area of passive IoT biomarker monitoring with significant potential for CVD monitoring. For example, chair-shaped systems that measure blood pressure biomarkers using similar technologies in smartwatch systems have been prototyped and tested [44]. Sleep monitoring using microwave based detection sensors placed under the bed sheet are advantageous in that they do not require contact with skin [45], while Gleichauf, et al. combined both microwave radar sensors and time-of-flight distance sensors for evaluating breathing rates in neonatal environments [46]. Pressure sensors have similarly been used to detect disordered breathing during sleep [47], and BCG signals have been used to assess sleep apnea [48]. Bed-integrated BCG sensors can also help detect cardiac arrhythmias [49] and have been explored for identifying reduced cardiac function in impending heart failure in an elderly patient [50]. Toilet seats with integrated ECG, PPG, and BCG sensors have been explored for accurate blood pressure, stroke volume, and blood oxygenation monitoring [51,52]. These systems aim to take advantage of frequent use and skin contact without requiring a wearable to collect longitudinal data for real-time CVD monitoring with future interests in heart failure patients. Toilets also open the possibility of passive urine testing and have already been explored within urinals [53]. As the urinalysis capabilities of these systems improve, broader CVD-monitoring applications arise. For example, the ability to measure urine sodium routinely not only may help assess CVD risk [16], but longitudinal profiling may provide prognostic information on pending heart failure compilations [54] as well as help assess treatment efficacy [55]. However, a recent clinical trial highlights the need for more research to reach the levels of consistency needed for clinical applications [56]. Though often more difficult with regards to signal analysis, an increasing use of deep learning models to analyze bio-signals from ECG, PPG, and BCG, instead of manually extracting features, are providing superior approaches to extracting personalized CVD signals in these increasingly demanding environments [57]. Table 1 summarizes these passive, dynamic biomarkers for CVD applications and their benefits and shortcomings.

## LEARNING HEALTH TRAJECTORIES OF GERIATRIC PATIENTS FROM LONGITUDINAL DATA

**Figure 2.**
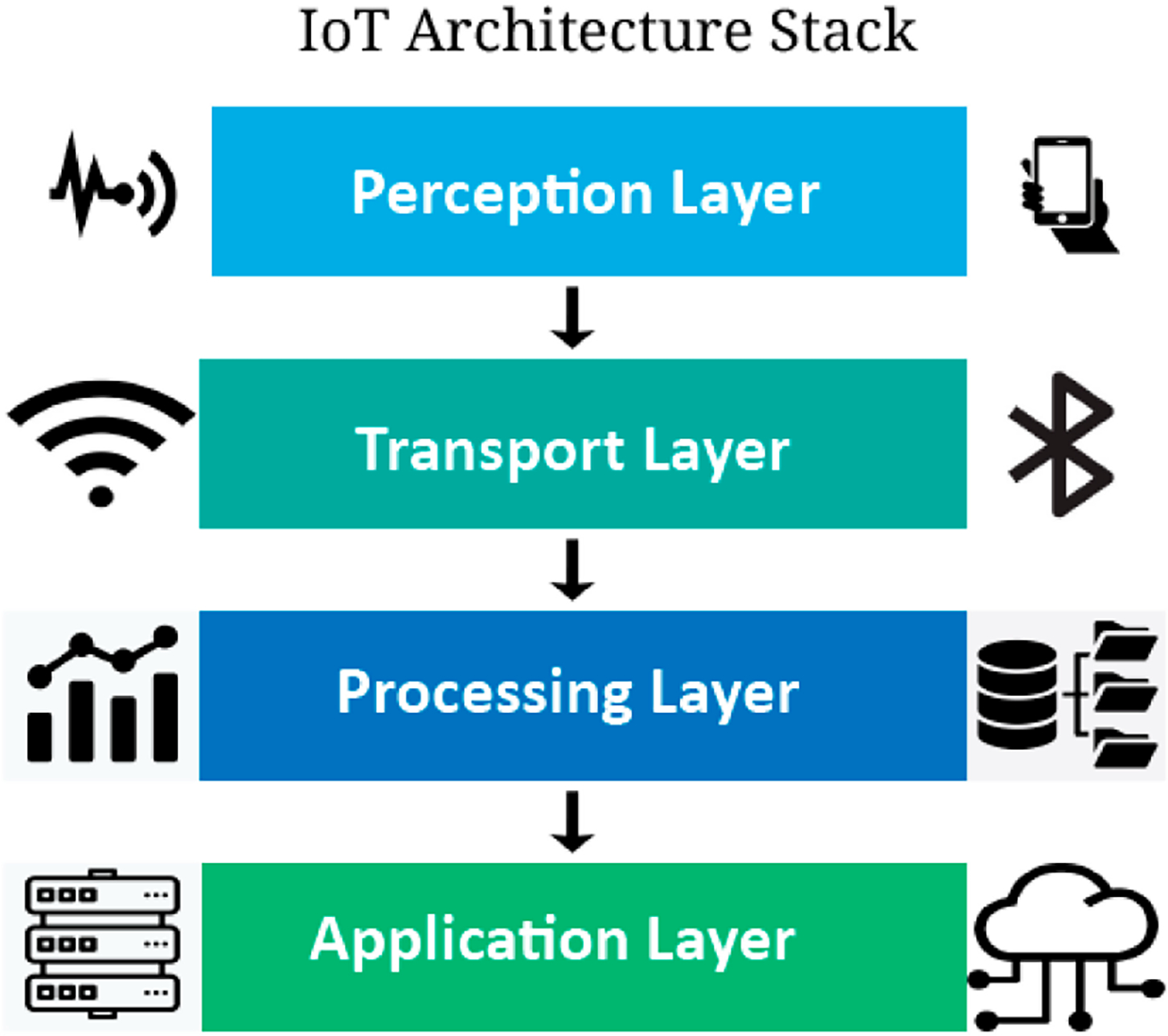
The 4-layer IoT architecture consists of the perception layer, transport layer, processing layer, and application layer.

In this section, we will explore the prevalent techniques used in analyzing longitudinal health trajectories of geriatric patients, which involves examining patient data during extended durations of time to identify patterns and forecast health trends. Within the context of CVD and hypertension prevention, we will discuss how artificial intelligence (AI) and ML techniques can be used to extract meaningful insights from observed data, and the implications of data security and ownership. This is typically viewed through a 4-layer IoT architecture model whereby the perception layer is responsible for collecting data from sensors and devices, the transport layer moves the data from the perception layer to the processing layer, the processing layer is where the data is analyzed and transformed into useful insights, which are then made available to end-users through the application layer (Figure 2). Additionally, we’ll consider the importance of user experience in designing health technology platforms that appropriately leverage user interfaces that enhance the tech adoption rate among elderly populations.

### Machine Learning and Artificial Intelligence

Integrating AI and ML with IoT has greatly impacted the way passive monitoring of older populations is conducted. AI and ML algorithms can analyze large amounts of data generated by wearable devices, sensors, and health records to identify patterns and predict the onset of disease [58]. This has led to increasingly more accurate and efficient monitoring that leads to earlier detection and prevention of chronic conditions. For example, the digital twin-enabled Twin Precision Treatment Program performed a 3 month study on 64 individuals using CGMs, Digital Twin technology, ML algorithms, and precision nutrition to aid treatment of patients with Type 2 diabetes which resulted in a significant reduction in blood pressure and a decrease in the percent of patients taking antihypertensive medications from 35.9% at baseline to 4.7% at 90 days [59]. Other applications can be viewed in Table 2. ML algorithms deployed on wireless, wearable ECG monitor data have been shown to be significantly accurate at automatically classifying cardiac anomalies among the elderly population [60]. The growth in these opportunities has largely stemmed from advancements in the processing power of computers that has allowed faster and more complex calculations on larger datasets [61]; widespread cloud computing services that enable efficient and safe storage and sharing of vast amounts of data [62]; widespread availability of high-speed internet connectivity [63]; widespread adoption of powerful mobile devices [64]; IoT devices used to monitor patients’ health in real-time and provide personalized recommendations and interventions [65]; and blockchain technologies working towards enabling authentic data sharing and interoperability across healthcare organizations [66]. These advancements have led to increased use of the following ML algorithms within IoT systems for tracking CVD biomarkers among the elderly:

- **Supervised learning:** This type of algorithm can be used to predict the onset of chronic diseases and heart issues based on patterns in physiological biomarker data that is pre-labeled before training [67]. For example, a comprehensive review published in the Computational and Structural Biotechnology Journal [68] presented models that use supervised learning algorithms (e.g., Random Forest, Naïve Bayes, Support vector machine, and Decision tree) to predict and assess heart failure in the adult population based on their HRV, blood pressure, and body mass index.
- **Unsupervised learning:** Unsupervised learning algorithms are more exploratory (e.g., K-means clustering). They can be used to identify patterns, clusters, and anomalies in *unlabeled* physiological biomarker datasets that may indicate the onset of a chronic disease or heart issue. In European Heart Journal - Digital Health [69], a review study presented analyses of unsupervised ML being used on 1693 patients hospitalized with Heart failure to reveal 6 disparate phenogroups common comorbidities in the older populations: coronary artery disease, valvular heart disease, atrial fibrillation, chronic obstructive pulmonary disease (COPD), obstructive sleep apnea (OSA), or few comorbidities.
- **Reinforcement learning (RL):** An RL algorithm learns to make decisions based on feedback from the environment. The algorithm interacts with the environment and learns by receiving rewards or penalties based on its actions [70]. The goal is to maximize the total reward over time. RL algorithms can be used to develop personalized treatment plans for elderly individuals based on their collected data sources. One example of RL used in practice by [71] showed how sedentary type 2 diabetic patients use data from their smartphone’s pedometer and to assist them in adhering to an exercise regimen that improved their glycemic control. RL algorithms in this scenario are able to learn over time and adjust the exercise plan to optimize the individual’s health outcomes.
- **Semi-supervised learning:** This type of algorithm combines elements of supervised and unsupervised learning and can be used to make predictions based on a combination of labeled and unlabeled data. Semi-supervised learning has proven to be useful in medical imaging analysis where data availability is often sparse (access to a large amount of unlabeled data, but a small amount of labeled data) [72].
- **Transformers**: Transformers are a type of deep learning algorithm designed to analyze and process large amounts of sequential data, such as natural language text and physiological biomarker data. Transformers use an attention mechanism to focus on different parts of the input sequence when processing each element in the sequence. This allows the model to capture long-range dependencies and relationships between words in a sentence or words in different sentences. Some well-known applications include language translation, question answering, and sentiment analysis. A study published in the IEEE Journal of Biomedical and Health Informatics [73] used a Transformer-based risk model to analyze electronic health records and subsequently provide explainability of predictions made for over 100,000 heart failure patients.

### User Experience (UX) and User Interface (UI)

The elderly population is particularly vulnerable to having difficulty adopting new technologies due to their age-related physical and cognitive impairments [80]. Reduced dexterity and vision quality can impair their ability to operate small interfaces and touch screens, while reduced hearing quality may hinder their ability to understand auditory cues and signals, such as emergency medical alerts. Thus, unfamiliar technologies like wearables are often disliked and viewed as cumbersome [81]. Additionally, older individuals face obstacles such as lower awareness of new technological advances, limited access to digital literacy support, and financial constraints that all make it difficult to keep up with modern trends in consumer tech and digital health advancements. Efforts in making passive monitoring more pervasive to alleviate the elderly’s interface issues must consider design practices and the consequences of human-computer interaction [82].

### Acquisition, Security, Ownership, and Safety of Big Data

With the rise of remote care services, connected devices, and other digital tools that can now be used in the home setting, there is a heightened need for adequate data protection for seniors who might not have the technical knowledge necessary to safeguard their protected health information. While it is possible for healthcare providers to ensure patient data is legally secured through HIPAA (Health Insurance Portability and Accountability Act) compliance regulations and other standards of responsible practice, there are still additional risks associated with using home health technologies that need to be addressed. For instance, if an elderly patient’s personal device is hacked or stolen then their sensitive medical information could become compromised. Additionally, if an elderly person transfers ownership of their technology device or equipment to someone else, either intentionally or unknowingly, then they could lose control over who has access to their medical history which can result in exploitations and medical identity theft [83]. Recent data breaches among consumer technologies, within digital health or otherwise, still hurt consumer trust for digital health technologies [8]. Only through proactive steps such as safeguarding patient information from unauthorized access, staff and patient education, audits, and well-defined breach protocols will patients and healthcare professionals be confident in ensuring optimal levels of security when utilizing home health technologies on behalf of senior citizens.

## CHALLENGES AND OPPORTUNITIES

Despite the many opportunities presented by IoT passive monitoring, there are also challenges that must be addressed. Ethical concerns related to data privacy and confidentiality remain one of the biggest challenges to IoT health data collection and use. Careful consideration must be given to protect sensitive patient data collected from unauthorized access and breaches.

Another challenge that must be addressed is the potential for bias in the data collected by IoT passive monitoring systems. The data collected by these systems can be affected by factors such as the type of device used, the location of the device, and the demographics of the patient population. If not carefully monitored and adjusted for, this bias can result in inaccurate and potentially harmful healthcare decisions. Thus, it is crucial for healthcare providers to carefully consider the limitations of IoT passive monitoring systems and develop methods to mitigate these biases. By addressing these challenges, healthcare providers can fully realize the benefits of IoT passive monitoring while ensuring patient privacy and safety.

IoT passive monitoring has improved healthcare and medical management for U.S. geriatric populations in a variety of ways that include, but not limited to early detection of chronic diseases, prompt medical assistance, telehealth visitations, and a reduction in human errors [84]. The trend towards increasing passivity among IoT health data collection is significant when it comes to compliance because the elderly population has barriers to independent use that can include impaired memory and decline in dexterity and sensory organs. Yet compliance is critical for the collection of longitudinal data necessary for the application of ML techniques to benefit CVD, including the ability to help stratify CVD risk, the ability to screen and diagnose for various CVD conditions, the ability to detect acute CVD events and alert caregivers, the ability to prognostically identify CVD trends and events, the ability to help personalize and manage chronic conditions and treatments, and even the ability to help motivate behavior changes that benefit CVD risk and disease treatment.

## RECOMMENDATIONS FOR FUTURE WORK

There are opportunities for advancing the field of IoT passive health monitoring by improving the breadth of physiological biomarker detection technologies. The majority of data collection is performed via smartwatches and other wearables that all use a handful of similar sensor modalities. Additional sensor modalities would provide richer data that could improve the accuracy of existing methods or create entirely new applications. Increasing passivity is another significant area of opportunity. For example, current approaches like smartwatches may improve battery performance to eliminate battery recharge burden, or more seamless integration of IoT sensors into furniture, clothing, and home appliances could create more zero-burden data collection platforms. There are significant opportunities in the application of ML algorithms for both novel digital biomarker discovery and better interpretation of health status and personalized health trajectories. The strength and relevance of ML algorithms come from the datasets they are built on and emphasizes the need for more data collection specifically from elderly populations. This will improve accuracy of models within these populations (Table 3).

The adoption of digital health tools such as telemedicine and wearables among the elderly U.S. population is a positive trend that demonstrates an increase in perceived value among the elderly and has significant potential to improve remote geriatric care either directly or through increased datasets used for improving current and future systems that will result in better personalized treatments.

## Figures and Tables

**Table 1.** Summary of passive, dynamic biomarkers under IoT exploration.

Biomarker Medium	Passive Sensor Technologies	IoT Integrations	Benefits	Shortcomings/Technical Barriers
Blood	Electrochemical; Spectroscopic	Continuous glucose monitors	A large variety of clinically validated biomarkers available	Still relatively bulky; frequent need to replace; direct skin contact; can still require direct contact with blood; limited validated biomarker detection platforms
Urine	Electrochemical; Spectroscopic	Toilets; urinals	Noninvasive; wide variety of validated biomarkers available; integration into act of daily living	Limited clinical validation of IoT integrated platforms; some integrated systems are viewed as too obtrusive
Heart rate and HRV	PPG; ECG; BCG	Watches; toilet seats; chairs; beds; clothing	Noninvasive; potentially seamless integration into daily living; several CVD applications	Most available approaches use watches which not everyone prefers; clothing and furniture integrations introduce more complexity in signal analysis
Pulse and blood pressure	PPG; ECG; BCG	Watches	Valuable CVD biomarkers and potential for increased testing compliance from passive testing framework	Blood pressure and blood pressure variability still require more clinical validation as signal processing is still largely in R&D

**Table 2.** Machine Learning Applications in IoT Platforms.

ML Category	ML Processing	Condition	Data Acquisition Platform (IoT application)	Data Input	Data Output
Supervised Learning [74]	K-Nearest Neighbor, Multilayer Perceptron, Linear-Support Vector Machine	Heart disease diagnoses	Patient data is deployed and stored on a cloud server	Body sensors	92.3% prognosis rate and 77.37% accuracy
Supervised Learning [75]	Support Vector Machines, Naïve Bayes, Random forest, Multi-layer perceptron	Hybrid recommender system for CVD	Wireless bio-sensor networks forwarding data to the cloud server	Heartbeat rhythm and ECG readings	Diagnose and classify 8 classes of CVD. Provides physical and dietary recommendations according to gender and age groups
Unsupervised Learning [76]	Density-based Spatial Clustering of Applications with Noise	Activity recognition monitoring	Time-based records of events	Daily behavioral and homecare sequence data	Detect the implicit irregularity of elderly health conditions
Reinforcement Learning [77]	Deep Q-network (DQN)	Lung cancer detection	Imaging classification of lung cancer	Pre-processed images	Lung tumor localization and treatment
Supervised Learning [78]	Random Forest, Gradient Boosting, K Nearest Neighbors, Support Vector Machine	Hypoglycemia detection system for diabetic patients	Glucose Sensor and Smart Watch	Heart rate, glucose, blood pressure, body temperature, shivering, and sweating	Real-time system alerts
Transformer [79]	Local Recurrent Transformer (LRT), Sentence BERT	Breathing abnormalities from physiological measurements (rate, pitch, depth)	Digital sound recorders	Breathing sounds	Prediction for breath sounds of the common cold, influenza, pneumonia, and bronchitis

**Table 3.** Potential discrepancies in longitudinal CVD biomarker correlations observed in the elderly.

Biomarkers	Technologies	Applications	Discrepancy identified between elderly and younger adults
HRV	ECG	Hypertension	Weaker correlation between longitudinal HRV and hypertension risk among older adults [39].
Glucose variability	Continuous glucose monitor	Hypoglycemia detection and glucose control	Different continuous glucose patterns, including higher mean CGM glucose, lower time-spent-in-range, and high rates of hypoglycemic values in nondiabetic elderly [85,86].
Longitudinal urine sodium	Electrochemical	Hypertension	Aging has been associated with reductions in renal sodium excretion, and correlations between urine sodium and blood pressure may only be significant in younger adults [87].
Blood pressure variability	Automatic electronic sphygmomanometer	CVD event prediction	While systolic blood pressure variability has been linked to stroke and coronary events in younger adults, some data suggests that in older subjects’ diastolic blood pressure variability is more strongly associated with coronary events and vascular or total mortality [88].
Sleep timing and sleep stage	Accelerometer; PPG	Sleep patterns	Age-related changes in circadian and homeostatic sleep drives may be accompanied by different cognitive and chronic disease risk effects from sleep deprivation in older adults [89,90].
Body temperature	Temperature sensor	Illness prediction	Blunted or dysfunctional thermoregulatory systems can alter body temperature response to illness [91].

## Data Availability

All data is available from the authors upon reasonable request.

## ABBREVIATIONS

AI: Artificial intelligence
BCG: Ballistocardiography
CVD: Cardiovascular disease
ECG: Electrocardiography
FDA: Food and Drug Administration
HIPAA: Health Insurance Portability and Accountability Act
HRV: Heart rate variability
IoT: Internet of things
ML: Machine learning
PPG: Photoplethysmography
RL: Reinforcement learning
